# Behavioural and psychological symptom determinants of carer burden across clinical syndromes associated with frontotemporal lobar degeneration and Alzheimer’s disease: a transdiagnostic analysis

**DOI:** 10.1007/s00415-026-14132-1

**Published:** 2026-09-12

**Authors:** Tao Chen, Qingyu Sun, David Foxe, Manisha Narasimhan, Claris Teng, Sau Chi Cheung, James Carrick, James R. Burrell, Yun Tae Hwang, Rebekah M. Ahmed, Olivier Piguet, Muireann Irish

**Affiliations:** 1 Brain and Mind Centre, The University of Sydney, Sydney, NSW 2050 Australia; 2 School of Psychology, The University of Sydney, Sydney, NSW Australia; 3 Central Clinical School, The University of Sydney, Sydney, NSW Australia; 4 Neuropsychology Unit, Royal Prince Alfred Hospital, Sydney, NSW Australia; 5 Macquarie University Health, Macquarie University, Sydney, NSW Australia; 6 Department of Neurology, Gosford Hospital, Gosford, NSW Australia; 7 School of Medicine and Public Health, Central Coast Clinical School, University of Newcastle, Newcastle, NSW Australia; 8 Department of Clinical Neurosciences, Memory and Cognition Clinic, Royal Prince Alfred Hospital, Sydney, NSW Australia

**Keywords:** Rigidity, Apathy, Motivation, Behavioural and psychological symptoms, Carer burden, Dementia

## Abstract

**Background:**

This study characterised overall transdiagnostic associations between psychological and behavioural symptoms and carer burden, and examined syndrome-specific contributions across clinically probable FTLD and AD groups.

**Methods:**

A total of 432 individuals with clinically probable FTLD and AD were included. The primary analytic cohort comprised 358 participants with complete carer burden data, including 230 and 128 individuals in the FTLD and AD diagnostic groups, respectively. Behavioural and psychological symptoms were assessed using the Cambridge Behavioural Inventory—Revised (CBI-R). Carer burden was measured with the Zarit Burden Interview (ZBI). Longitudinal CBI-R and ZBI data were analysed in 172 participants with available ZBI data at both baseline and follow-up (mean interval ≈ 1 year).

**Results:**

Behavioural rigidity (standardised *β* = 0.15, 95% CI [0.03, 0.28], *p* = 0.015) and apathy (standardised *β* = 0.14, 95% CI [0.02, 0.26], *p* = 0.020) showed the strongest associations with carer burden overall. Behavioural rigidity was salient in the FTLD group (standardised *β* = 0.18, 95% CI [0.02, 0.33], *p* = 0.024), whereas apathy (standardised *β* = 0.26, 95% CI [0.04, 0.47], *p* = 0.021) and abnormal behaviour (standardised *β* = 0.26, 95% CI [0.05, 0.48], *p* = 0.018) were salient in the AD group. Longitudinal increases in mood-related symptoms (standardised *β* = 0.24, 95% CI [0.001, 0.48], *p* = 0.048) predicted greater carer burden at follow-up.

**Conclusions:**

Our findings highlight the relevance of symptom-focussed strategies for managing carer burden across dementia syndromes. Group-specific analyses suggest prioritising behavioural rigidity in FTLD syndromes and apathy and abnormal behaviour in AD subtypes, while timely management of changes in mood symptoms may help alleviate carer burden over time.

**Supplementary Information:**

The online version contains supplementary material available at https://doi.org/10.1007/s00415-026-14132-1.

## Introduction

Dementia is associated with substantial physical, financial and socioemotional impact, much of which is borne by family carers who provide day-to-day support [1–3]. With disease progression, carer burden becomes a critical clinical outcome, closely linked to carer mental health, increased health service utilisation, and premature institutionalisation of the individual with dementia [1, 4].

Behavioural and psychological symptoms of dementia (BPSD) are the primary factors contributing to carer burden [5, 6]. Previous studies using the Neuropsychiatric Inventory (NPI) [7] have shown that an array of symptoms including disinhibition, apathy, aberrant motor behaviours, agitation, sleep disturbances, and psychosis are associated with carer burden and distress in frontotemporal lobar degeneration (FTLD) [8–11]. Emotional disturbances (e.g. apathy and depression/dysphoria), delusions, and behavioural problems, like disinhibition and abnormal motor behaviours, are more commonly associated with carer burden and distress in Alzheimer’s disease (AD) [10–12]. Crucially, global cognition does not appear to be a predictor of carer burden in either AD or FTLD [8, 10]. This suggests that efforts to reduce carer burden in dementia should prioritise alleviating BPSD. Identifying which BPSDs most strongly drive carer burden represents the critical first step to effective behavioural management and carer support.

Despite their clinical relevance, the behavioural drivers of carer burden remain poorly characterised. One limitation is the narrow behavioural coverage of commonly used clinical instruments, which can fail to capture behavioural problems frequently reported as taxing by carers. One such behavioural domain is that of rigid and repetitive behaviours, which are particularly salient in FTLD [13, 14] and also reported in AD [15]. Repetitive behaviours include stereotypies (e.g. excessive hand rubbing), compulsions (e.g. excessive hand washing), stereotyped language (e.g. catchphrases), impulsive behaviours (e.g. pathological gambling), hoarding, restricted interests, insistence on sameness, and ritualistic actions [16]. Failure to consider such behaviours and their impact on caregiving demands results in an incomplete picture of carer burden in dementia from current empirical models [8, 10]. In addition, the clinical importance of specific symptoms and their relative impact on the carer remains poorly understood. In real-world care settings, clinicians and carers prioritise the symptoms requiring the most urgent management, particularly when caregiving resources, clinical contact time, and treatment options are limited. However, empirical evidence to guide such prioritisation remains relatively scarce.

A further consideration is that carer burden fluctuates over the disease course as different symptoms ebb and flow. Carers may adapt and develop successful coping mechanisms or strategies to manage certain behavioural features that build into their routine; some BPSDs may subside over time, while other emergent or unanticipated behaviours may precipitate acute distress. Previous work in FTLD has characterised the multidimensional nature and prognostic relevance of behavioural symptoms [17, 18], while more recent longitudinal evidence demonstrates that behavioural symptom profiles evolve over time and differ across diagnostic groups [19]. However, less is known about how within-person changes in behavioural and psychological symptoms relate to concurrent changes in carer burden. Understanding these dynamic symptom–burden relationships may provide important insights into how evolving symptom profiles affect carers over time.

To address these gaps in the literature, the present study aimed to identify behavioural determinants of carer burden at multiple levels of inference using both cross-sectional and longitudinal approaches. First, we examined behaviour–burden associations leveraging the broad behavioural coverage of the Cambridge Behavioural Inventory—Revised (CBI-R) [20] to capture dimensions commonly reported by carers as disruptive in their daily lives. Next, we quantified the relative contributions of distinct symptom dimensions across a combined dementia cohort to characterise overarching behavioural and psychological drivers of carer burden. To understand disease-specific drivers of carer burden, we then evaluated these associations separately within AD and FTLD clinical groups. Finally, we conducted exploratory longitudinal analyses to examine associations between changes in BPSD and level of carer burden over time. In doing so, this study refines current models of behavioural impairment and carer burden and provides a foundation for targeted intervention approaches tailored to the dementia subtype and disease staging of the individual.

### Methods

#### Patients

A total of 432 individuals diagnosed with dementia through the FRONTIER research clinic at the Brain and Mind Centre, University of Sydney, Australia, between 2008 and 2025 were included in this study. Based on clinical diagnoses, participants were classified into clinically probable FTLD (*n* = 277) and AD (*n* = 155) diagnostic groups. Specifically, the FTLD group included 97 participants with behavioural variant frontotemporal dementia (bvFTD), 49 with left-sided semantic dementia (SD-left), 23 with right-sided semantic dementia (SD-right), 44 with progressive nonfluent aphasia (PNFA), 29 with progressive supranuclear palsy (PSP), and 35 with corticobasal syndrome (CBS). The AD group included 110 individuals with typical AD and 45 participants with logopenic progressive aphasia (LPA).

Dementia diagnoses were determined according to internationally recognised diagnostic criteria by a multidisciplinary team comprising a senior neurologist, neuropsychologist, and speech pathologist. Diagnostic consensus was reached using the relevant criteria at the time of assessment (AD [21]; bvFTD [22]; PNFA, SD, LPA [23]; PSP [24]; CBS [25]). All patients achieved a minimum score of 40/100 on the Addenbrooke’s Cognitive Examination—III (ACE-III) [26] at baseline. Exclusion criteria included a history of psychiatric illness, significant head injury with loss of consciousness, alcohol or substance misuse, or insufficient English proficiency to complete assessments.

Ethical approval was obtained from the South Eastern Sydney Local Health District, University of New South Wales, and University of Sydney Human Research Ethics Committees. Written informed consent was provided by all participants or their person responsible in accordance with the Declaration of Helsinki.

Participants were seen at the FRONTIER clinic on two occasions: (i) baseline and (ii) follow-up visit, which occurred approximately 1 year after baseline (i.e. 0.5–1.5 years).

#### Carer burden

Carer burden was assessed with the Zarit Caregiver Burden Interview (ZBI) [27]. This 12-item instrument captures the emotional, physical, and role-related demands of caregiving. Carers rate the frequency of burden-related experiences (e.g. stress balancing caregiving with other responsibilities, health consequences, and embarrassment about the patient’s behaviour) on a 0–4 Likert scale (*0* = never to *4* = nearly always). Total scores range from 0 to 48, with higher values denoting greater burden.

#### Behavioural and psychological symptoms

Behavioural and psychological symptoms over the preceding month were measured using the carer-reported Cambridge Behavioural Inventory—Revised (CBI-R) [28]. Seven of the 10 original CBI-R subdomains were included: abnormal behaviour (items 18–23), mood (items 24–27), abnormal beliefs (items 28–30), eating habits (items 31–34), sleep (items 35–36), stereotypic and motor behaviours (items 37–40), and motivation (items 41–45). All items within each selected subdomain were retained without modification or reassignment. The remaining three subdomains (memory and orientation, everyday skills, and self-care) were not included as they primarily assess cognitive and functional impairment rather than behavioural and psychological symptoms. For brevity, stereotypic and motor behaviours are referred to throughout as behavioural rigidity, while apathy refers specifically to symptoms captured by the CBI-R motivation subscale. Carers rated the frequency of each symptom on a five-point Likert scale (*0* = never to *4* = constantly), with higher scores reflecting greater symptom frequency.

#### Global cognition and other assessments

Global cognition was measured with the Addenbrooke’s Cognitive Examination—Revised (ACE-R) [29] or its more recent iteration, the Addenbrooke’s Cognitive Examination—III (ACE-III) [26], which assess attention/orientation, memory, fluency, language, and visuospatial abilities. To harmonise cognitive scores across participants, ACE-R results were converted to ACE-III equivalents using validated algorithms [30]. Disease duration was derived as the interval between first reported symptom onset and the date of cognitive assessment, while disease severity was quantified using the Frontotemporal Dementia Functional Rating Scale (FRS) [31].

#### Statistical analyses

Of the 432 participants included in the study, 358 had complete carer burden (ZBI) data and constituted the primary analytic cohort. Across the seven BPSD domains, the proportion of missing observations ranged from 0 to 3.1% in the AD group and from 0.4 to 4.3% in the FTLD group (Supplementary Table 1). Missing clinical and cognitive data within this 358-participant cohort were handled using multiple imputation by chained equations, with predictive mean matching (PMM) implemented in the MICE package [32]. The distribution of missing data across variables is presented in Supplementary Fig. 1. Multivariate linear regression models were used to evaluate the extent to which baseline BPSD and global cognition predicted baseline carer burden in the overall dementia cohort, as well as within the FTLD and AD groups separately. Longitudinal changes in carer burden from baseline to follow-up were modelled in relation to corresponding changes in BPSD, first in the full cohort, and then within FTLD and AD groups separately. To assess the consistency of associations across diagnoses, diagnosis-specific scatterplots with fitted regression lines were generated and used to visualise association patterns. All models were adjusted for baseline patient age, sex, education, disease duration, disease severity, and diagnostic category. To quantify the reliability and relative influence of each predictor, we report standardised regression coefficients with 95% confidence intervals (CIs) and corresponding relative importance metrics using the relaimpo package [33]. Relative importance was estimated using the LMG (Lindeman–Merenda–Gold) metric. LMG values were normalised to sum to 1 across all predictors within each model, representing the proportion of model-explained variance allocated to each predictor. Relative importance values do not have established cut-off thresholds and were therefore interpreted on a relative, comparative basis, with their magnitude considered in relation to the observed range within each model. Combining standardised regression coefficients and relative importance analyses allows complementary characterisation of predictors, whereby standardised regression coefficients estimate independent associations with carer burden, while relative importance metrics capture each predictor’s contribution to explained variance while accounting for correlations with other symptoms. This combined approach enables the identification of both independent effects and shared, synergistic contributions among co-occurring behavioural and psychological symptoms.

## Results

### Background information of patients

Table 1 and Supplementary Table 2 present the demographic and clinical characteristics of the dementia groups included in the primary analyses (*N* = 358). Detailed breakdown of demographic information for carers is provided in Supplementary Table 3.

**Table 1:** Demographic and clinical information for dementia samples

	Full dementia cohort (*n* = 358)	FTLD group^a^ (*n* = 230)	AD group^b^ (*n* = 128)
Age, years	65.89 (8.08)	65.94 (8.15)	65.82 (7.96)
Education, years	12.25 (3.08)	12.07 (3.06)	12.59 (3.11)
Sex, male:female	202:156	129:101	73:55
Disease duration, years	3.75 (2.39)	4.04 (2.53)	3.24 (2.02)
ACE-III [100]	71.03 (13.89)	72.91 (14.04)	67.65 (13.06)
FRS logit score [− 6.66, 5.39]^c^	0.99 (1.83)	0.89 (1.89)	1.17 (1.71)
CBI-R Total [180]^d^	46.06 (29.93)	48.89 (31.68)	40.97 (25.20)

Means with standard deviations in parentheses. Maximum test score or score range provided in square brackets, where appropriate. *ACE-III* the Addenbrooke’s Cognitive Examination—Third edition, *CBI-R* Cambridge Behavioural Inventory-Revised, *FRS* Frontotemporal Dementia Functional Rating Scale, *FTLD* clinically probable frontotemporal lobar degeneration, *AD* clinically probable Alzheimer’s disease

^a^FTLD group comprises behavioural variant frontotemporal dementia (bvFTD), left-sided semantic dementia (SD-left), right-sided semantic dementia (SD-right), progressive nonfluent aphasia (PNFA), corticobasal syndrome (CBS), and progressive supranuclear palsy (PSP). ^b^AD group comprises typical AD and logopenic progressive aphasia (LPA). ^c^Lower logit scores, as derived using Rasch analyses, denote greater levels of functional impairment on the FRS. ^d^Higher scores on the CBI-R denote greater behavioural disturbances

### Multivariate regression models across the full dementia cohort and FTLD and AD groups at baseline

**Fig. 1:**
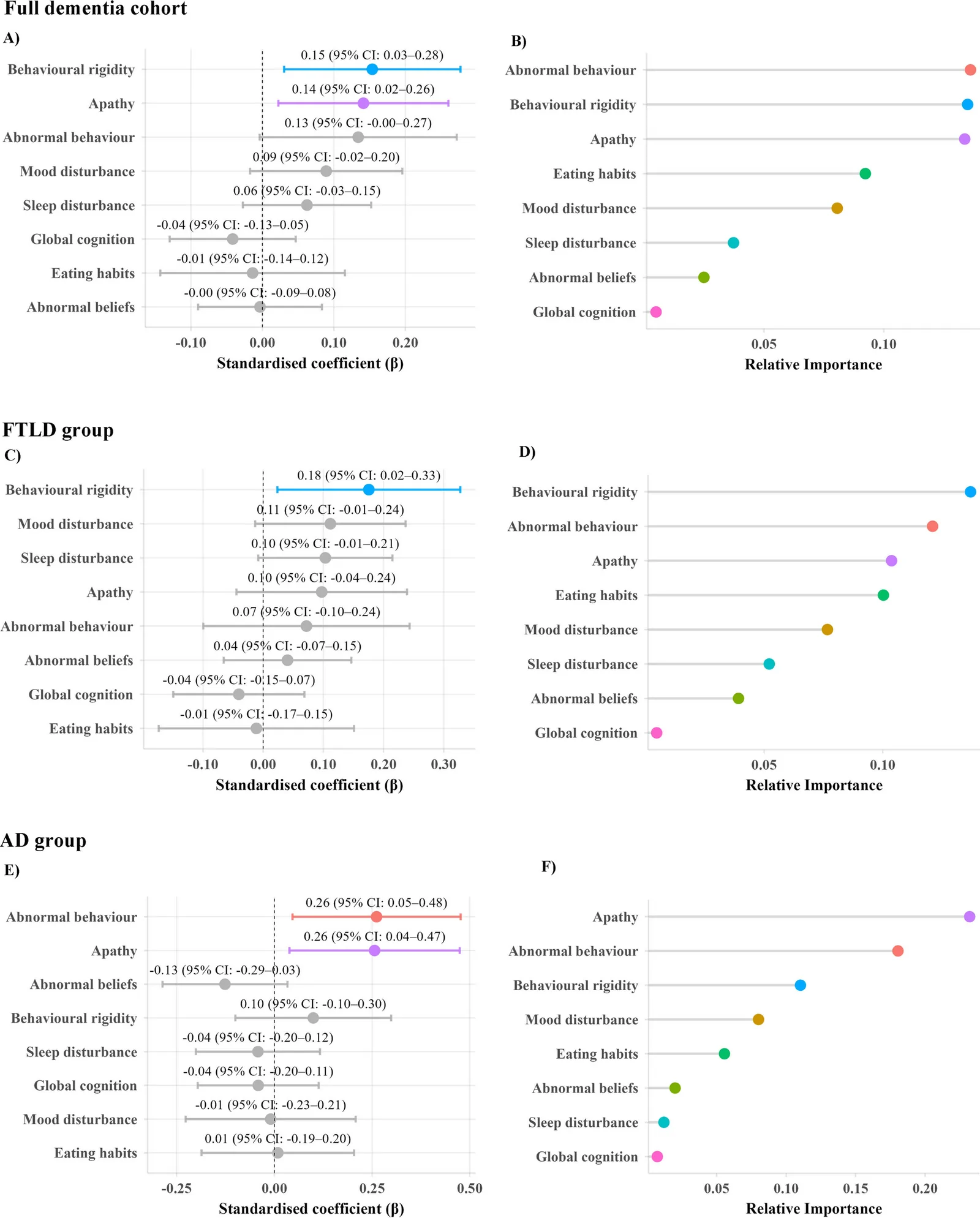
Associations between BPSD (assessed using the CBI-R), global cognition (assessed by ACE-III), and carer burden (assessed by ZBI) at baseline in the primary analytic cohort (*N* = 358), adjusting for patient age, sex, education, disease duration, disease severity, and diagnostic category. Standardised regression coefficients from multivariate linear regression models predicting carer burden are shown for **A** the full dementia cohort, **C** the FTLD group, and **E** the AD group. Relative importance values from the same models are shown for (**B**) the full dementia cohort, **D** the FTLD group, and **F** the AD group. Floating lines depict 95% confidence intervals (CIs), and an association is supported when the CI does not include zero. Higher relative importance value represents larger importance in the regression model. Lower ACE-III scores reflect poorer global cognition, while higher CBI-R and ZBI scores indicate greater symptom severity. CBI-R, Cambridge Behavioural Inventory-Revised; ACE-III, the Addenbrooke’s Cognitive Examination—Third edition; ZBI, the Zarit Caregiver Burden Interview. FTLD, clinically probable frontotemporal lobar degeneration; AD, clinically probable Alzheimer’s disease. The FTLD group comprised clinical syndromes typically associated with FTLD pathology, including behavioural variant frontotemporal dementia (bvFTD), left-sided semantic dementia (SD-left), right-sided semantic dementia (SD-right), progressive nonfluent aphasia (PNFA), corticobasal syndrome (CBS), and progressive supranuclear palsy (PSP). The AD group comprised clinical syndromes typically associated with AD pathology, including typical AD and logopenic progressive aphasia (LPA)

Multiple regression models were run to explore the relative importance of each BPSD symptom measured by the CBI-R, including apathy as indexed by the CBI-R Motivation subscale, as well as global cognition measured by the ACE-III, in predicting carer burden on the ZBI (see Fig. 1). First, we looked across the full dementia cohort (*n* = 358) controlling for patient age, sex, education years, disease duration, disease severity and dementia subtype. Two symptoms were found to significantly positively predict carer burden across the full dementia cohort, namely behavioural rigidity (standardised *β* = 0.15, 95% CI [0.03, 0.28], *p* = 0.015) and apathy (standardised *β* = 0.14, 95% CI [0.02, 0.26], *p* = 0.020). Relative importance values ranged from 0.005 to 0.136 across the domains of interest, with abnormal behaviour showing the largest contribution (0.136), followed by behavioural rigidity (0.135) and apathy (0.134). Importantly, global cognition as measured by the ACE-III (*p* = 0.355) and other BPSDs assessed by the CBI-R did not significantly predict carer burden (all *p* values > 0.05). In the combined patient cohort, Supplementary Fig. 2 demonstrates broadly consistent directional patterns of association for the two BPSD domains in each diagnostic group, suggesting that the observed associations reflect a transdiagnostic pattern rather than diagnosis-specific effects.

Next, we ran the multiple regression model in the clinically probable FTLD group (*n* = 230) and found that carer burden was significantly predicted by behavioural rigidity (standardised *β* = 0.18, 95% CI [0.02, 0.33], *p* = 0.024). Relative importance values ranged from 0.005 to 0.137 across domains of interest, with behavioural rigidity showing the largest contribution (importance score = 0.137). Again, global cognition on the ACE-III (*p* = 0.466) and other BPSDs on the CBI-R did not predict carer burden (all *p* values > 0.05). Across FTLD clinical subtypes, Supplementary Fig. 3 illustrates broadly consistent directional patterns of association, suggesting that the observed associations are not driven by any single FTLD subtype.

Finally, for the AD group (*n* = 128), two BPSD symptoms significantly predicted carer burden, namely apathy (standardised *β* = 0.26, 95% CI [0.04, 0.47], *p* = 0.021) and abnormal behaviour (standardised *β* = 0.26, 95% CI [0.05, 0.48], *p* = 0.018). Relative importance values ranged from 0.007 to 0.232 across the domains of interest, with apathy showing the largest contribution (0.232), followed by abnormal behaviour (0.180). Crucially, global cognition (*p* = 0.599) and all other BPSD subtypes failed to predict carer burden within the AD group (all *p* values > 0.05), and the observed associations were not found to be driven by any single AD clinical subtype (Supplementary Fig. 4).

In summary, behavioural rigidity emerged as the strongest and most important predictor of carer burden for the FTLD group, while the non-cognitive symptom of apathy emerged as the most important predictor of carer burden in the AD group.

### Prediction of longitudinal change in carer burden

Next, we explored how changes in BPSD symptoms from T1 to T2 (mean time interval ~1 year) related to changes in carer burden over time (ZBI T1 to T2). We calculated the relative importance of longitudinal change in each behavioural and psychological symptom and change in carer burden across the combined patient cohort and in each group separately. Separate multiple regression analyses were conducted, with longitudinal changes in each BPSD domain from T1 to T2 entered as predictors and longitudinal change in ZBI from T1 to T2 as the outcome, controlling for baseline patient age, sex, education years, illness duration, disease severity and diagnosis factor.

**Fig. 2:**
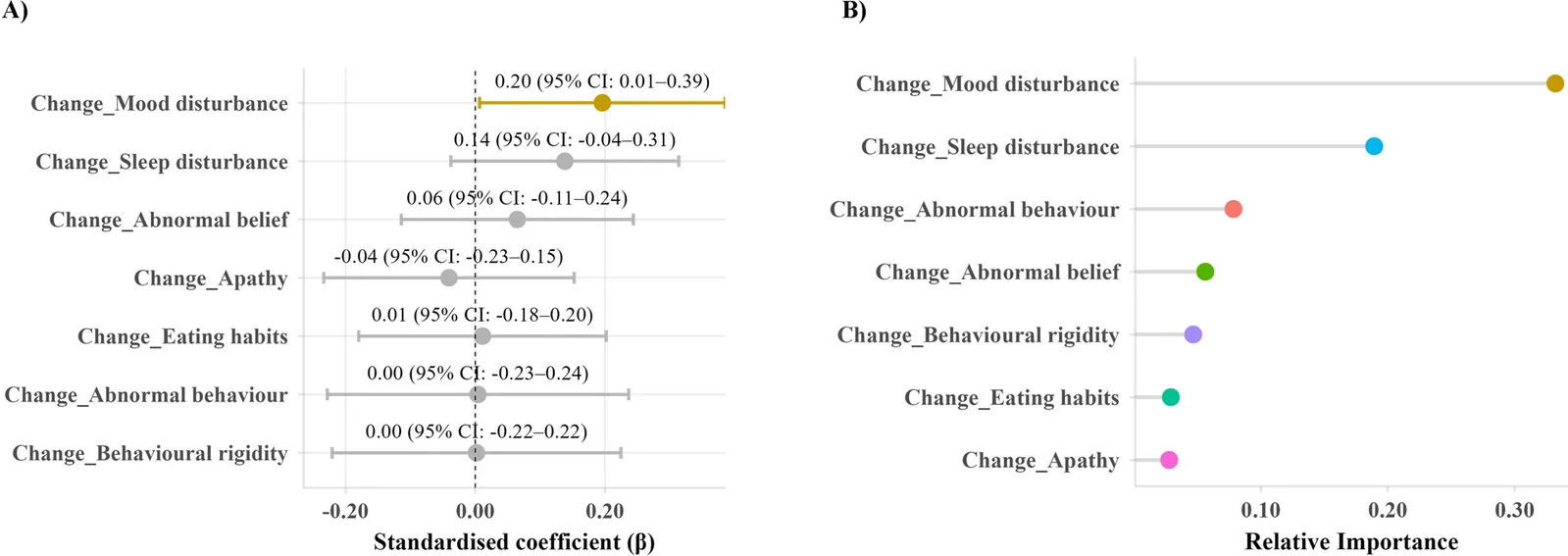
Associations between longitudinal changes (i.e. T2-T1) in BPSD (assessed by CBI-R) and longitudinal changes (i.e. T2-T1) in carer burden (assessed by ZBI) in participants with complete ZBI data at both time points (*N* = 172), adjusting for baseline patient age, sex, education, disease duration, disease severity, and diagnostic category. Standardised regression coefficients from multivariate linear regression models predicting longitudinal change in carer burden are shown for **A** the full dementia cohort. Relative importance values from the same models are shown for **B** the full dementia cohort. Floating lines depict 95% confidence intervals (CIs) and an association is supported when the CI does not include zero. Higher relative importance values represent stronger contributions to the regression model. *CBI-R* Cambridge Behavioural Inventory–Revised, *ZBI* Zarit Caregiver Burden Interview

Across the full dementia cohort (*n* = 172), longitudinal change in mood (e.g. irritability and agitation) was the only symptom to significantly positively predict longitudinal change in carer burden (standardised *β* = 0.20, 95% CI [0.01, 0.39], *p* = 0.043) and showed the largest relative importance (importance score = 0.332) (see Fig. 2). In contrast, no significant longitudinal predictors of carer burden were identified within either the FTLD or AD groups. The overall patterns of association between longitudinal changes in BPSD and carer burden across the entire longitudinal cohort are shown in Supplementary Fig. 5.

### Sensitivity analysis

Sensitivity analyses using datasets with imputed clinical and cognitive data, including ZBI, yielded consistent results in both cross-sectional (Supplementary Fig. 6) and longitudinal analyses (Supplementary Fig. 7). To further assess the robustness of the cross-sectional FTLD-associated findings to diagnostic composition, additional sensitivity analyses excluded bvFTD and, separately, semantic dementia (left and right SD). Behavioural rigidity remained the strongest predictor of carer burden in both analyses, indicating that the finding was not driven by either clinical subtype (Supplementary Fig. 8).

## Discussion

This study investigated transdiagnostic and syndrome-specific associations between BPSD and carer burden in clinical variants of FTLD and AD and examined how these relationships evolve over time. At baseline, behavioural rigidity and apathy were significantly associated with carer burden, with behavioural rigidity playing the most prominent role in the FTLD group, whereas apathy, as captured by the CBI-R Motivation subscale, and abnormal behaviour were most prominent in the AD group. Over time, however, longitudinal changes in mood-related symptoms (e.g. agitation and irritability) emerged as the sole predictor of increased carer burden transdiagnostically. Our findings point to multifaceted drivers of carer burden in dementia, some of which occur regardless of the dementia type while others appear to be disease specific. This study provides novel information regarding the dynamic interplay between BPSDs and carer burden over time, highlighting the need for targeted supports that are tailored to the person based on diagnostic subtype and disease staging.

A major finding from this study is of common and unique predictors of carer burden in FTLD and AD groups. Using the CBI-R, we replicated associations between apathy, disinhibition and increased carer burden, as traditionally captured by the NPI [8, 10]. Crucially, we demonstrate for the first time, that behavioural rigidity shows a robust and consistent association with carer burden across dementia syndromes, with particularly pronounced effects in FTLD as indexed by standardised regression coefficients and relative importance value. This finding is notable given that disinhibition is commonly associated with carer burden in FTLD, presumably through challenging behaviours that can be socially inappropriate, disruptive, or have real-world consequences (e.g. financial loss and legal implications) [8, 11]. Behavioural rigidity, however, is a core diagnostic feature of bvFTD, with emerging evidence pointing to its presence in both right- and left-sided SD [13, 16, 34, 35]. Such rigid behaviours are generally of high frequency and resistant to change, imposing constraints on the timing, sequencing, and nature of routine activities. As a result, caregiving may become organised around the need to accommodate or navigate ritualistic behaviours and rules at the expense of efficiency or shared decision-making [36]. At their extreme, rigid schedules may extend beyond an increase in caregiving workload to a more fundamental reconfiguration of carers’ daily lives. We tentatively suggest that over time, rigid behaviours generate strain on carers leading to their disproportionate impact on carer burden relative to other behavioural and psychological symptoms. Notably, behavioural rigidity remained the strongest predictor after excluding bvFTD participants, suggesting that its relevance to carer burden extends beyond behaviourally predominant presentations. However, its contribution may vary across the disease course, reflecting differences in the timing and prominence of behavioural symptoms across FTLD clinical syndromes. Such heterogeneity should be considered when interpreting the pooled FTLD findings.

In the combined AD group, apathy emerged as one of the most significant predictors of carer burden, consistent with previous findings [10, 12, 37]. A previous study using interpretative phenomenological analysis suggests that apathy may contribute to carer burden through the reconfiguration of caregiving roles and persistent emotional conflict [38]. In this context, the authors proposed that diminished motivation in AD may shift responsibility for initiating and sustaining everyday activities onto carers. Carers are consequently required to navigate the tension between facilitating engagement, often by subtly steering decisions, and preserving the person’s autonomy, a process that can lead to frustration, guilt, and the gradual acceptance of diminished reciprocal involvement.

We highlight the point that global cognition was not associated with carer burden in either AD or FTLD groups, indicating that cognitive severity alone provides limited guidance for burden management. This dissociation is particularly informative in AD, where cognitive decline is often assumed to be the primary driver of carer distress. Our findings instead highlight the central role of motivational disturbances in shaping carers’ daily demands and emotional strain, independently of global cognitive severity. Consequently, efforts to reduce carer burden should extend beyond cognitive staging to prioritise the assessment and management of BPSD in routine care, underscoring the importance of psychoeducation at diagnosis, particularly in AD, to prepare carers for apathy as a prominent and burdensome feature.

As a secondary exploratory analysis, we examined the relationship between changes in BPSD and carer burden over time. Constraining our focus to a 1-year follow-up period, changes in mood-related symptoms (e.g. irritability and agitation) emerged as the prominent correlate of changes in carer burden at the transdiagnostic level. Changes in mood-related symptoms are more likely to vary with situational demands over time and may be particularly impactful if difficult to anticipate in the course of caregiving. Indeed, unpredictable and uncontrollable events have been shown to predict higher levels of negative affect (e.g. feelings of desperation and tension) in carers of individuals with dementia [39]. In caregiving contexts, such unpredictability may require carers to modify previously effective strategies and reallocate time and emotional resources. Importantly, disruptive mood-related behaviours such as agitation/aggression of individuals with dementia, even when infrequent, are associated with disproportionately high levels of carer stress [40], highlighting the emotional cost of repeatedly adapting to such behaviours. Nevertheless, these exploratory longitudinal findings should be interpreted in the context of substantial differences in clinical progression across diagnostic syndromes. The contribution of behavioural symptoms to carer burden may vary across the disease course, and pooled associations may therefore not fully capture syndrome-specific differences in the magnitude or direction of symptom–burden relationships. Although diagnostic syndrome was accounted for in the analyses, further characterisation of these longitudinal patterns will require larger syndrome-specific samples.

By combining standardised regression coefficients with relative importance rankings, we propose a differentiated interpretation of behavioural and psychological symptoms in relation to carer burden. Symptoms characterised by both significant regression coefficients and high relative importance may warrant prioritisation as direct intervention targets (e.g. behavioural rigidity in FTLD clinical variants). In contrast, symptoms with non-significant regression coefficients but high relative importance (e.g. abnormal behaviour in FTLD subtypes) may reflect shared contributions across co-occurring behaviours, suggesting that effective reduction of carer burden is more likely to require integrated management strategies targeting multiple interacting symptoms rather than isolated interventions.

While this study provides new insights into the antecedents of carer burden in dementia, several limitations should be acknowledged. This study aimed to examine the relative contribution of individual BPSD to carer burden in dementia and therefore focussed primarily on controlling for patient-related factors, including demographic, cognitive, and disease characteristics. Importantly, however, carer burden is also shaped by carer characteristics, such as personality, coping style, social support, psychological well-being, and time spent providing care, all of which have been shown to influence perceived burden [41–43]. Further contextual factors, including low household income and limited access to governmental support, may exacerbate carer burden [41]. Future studies adopting a more comprehensive framework that integrates patient symptoms, carer characteristics, and contextual factors will be essential to fully characterise the determinants of carer burden. Moreover, it is highly likely that trajectories of carer burden differ by FTLD subtypes [8]. Our sample size precluded reliable group analyses within individual diagnostic categories (e.g. PSP, SD, and PNFA). To address this limitation, we visually examined whether the associations between BPSD and carer burden showed consistent patterns across diagnostic groups and explicitly controlled for diagnosis as a covariate in regression analyses of the full dementia cohort and within the FTLD and AD groups. These approaches support the robustness of our transdiagnostic findings, although group-specific analyses may likely provide additional insight into syndrome-specific relationship patterns. Future studies with larger, well-characterised samples, potentially through multi-centre collaborations, will be needed to delineate these subtype-specific associations more precisely. Furthermore, to maximise data and guard against study attrition, we focussed our longitudinal analyses on approximately 1-year follow-ups, which also helped minimise confounding effects arising from widely variable inter-assessment intervals. Previous work has linked behavioural disturbances, including impulsivity and apathy, to reduced functionally independent survival across FTLD syndromes, even after excluding bvFTD [44], highlighting the broader clinical significance of behavioural symptoms as disease progresses. Future studies incorporating extended follow-up periods are needed to clarify if changes in specific behavioural symptoms relate to carer outcomes. Pathological confirmation of disease was not available for this cohort and as such, we stress the need for future studies incorporating these data to validate the present findings. Finally, the FRS was used as an approximate measure of overall functional disease severity rather than a diagnosis-specific measure of disease progression and has not been specifically validated across all diagnostic syndromes included in this study. This limitation should be considered when interpreting analyses adjusted for disease severity.

Our findings have important implications for the clinical management of carer burden in dementia. Given the prominent cross-sectional associations of behavioural rigidity and apathy with carer burden across syndromes, routine screening and management of these behavioural features should be considered in all patients with dementia. These cross-sectional findings highlight behavioural features that contribute to carer burden and may help guide the prioritisation of long-term management strategies. When multiple BPSD are present, behavioural rigidity emerges as warranting particular attention in FTLD clinical syndromes, and apathy and abnormal behaviour in AD clinical subtypes. Importantly, our longitudinal analyses revealed that longitudinal changes in carer burden were primarily driven by fluctuations in mood-related symptoms (e.g. irritability and agitation) across syndromes, underscoring the role of affective symptoms as proximal and dynamic drivers of burden over time. This pattern suggests that, beyond managing stable behavioural features, ongoing monitoring and timely management of mood-related changes may be critical to prevent escalations in carer burden.

Currently, in the absence of effective disease-modifying therapies, symptom-focussed management may offer the greatest potential for reducing carer burden [45–47]. Given recognised neurotransmitter system dysfunctions in FTLD, particularly involving serotonergic and dopaminergic systems [48–50], and their links to cognitive flexibility [51] modulation of serotonergic pathways may help alleviate behavioural symptoms such as rigidity and repetitive behaviours in dementia. Importantly, with due consideration of safety, non-pharmacological approaches should be prioritised when symptoms are not severe or pose minimal risk, consistent with current clinical recommendations [6, 52].

## Supplementary Information

Below is the link to the electronic supplementary material.

Supplementary file1 (DOCX 2235 KB)

## Data Availability

All human subjects provided informed consent. The data that support the findings of this study are available on request from the corresponding author. The data are not publicly available due to privacy or ethical restrictions.
