# Supplementary material for: Behavioural and psychological symptom determinants of carer burden across clinical syndromes associated with frontotemporal lobar degeneration and Alzheimer’s disease: a transdiagnostic analysis
Source: J Neurol. 2026 Sep 12;273(10):592. doi: 10.1007/s00415-026-14132-1 (PMC13570929; doi:10.1007/s00415-026-14132-1)
Supplement: Supplementary file 1 [file 415_2026_14132_MOESM1_ESM.docx]

**SUPPLEMENTARY MATERIAL**

**Behavioural and psychological symptom determinants of carer burden across clinical syndromes associated with frontotemporal lobar degeneration and Alzheimer’s disease: A transdiagnostic analysis**

Tao Chen^1,2^, Qingyu Sun^1,2^, David Foxe^1,2^, Manisha Narasimhan^1,2^, Claris Teng^1,2^, Sau Chi Cheung^1,2,4^, James Carrick^1,2^, James R. Burrell^5^, Yun Tae Hwang^1,6,7^, Rebekah M. Ahmed^1,8^, Olivier Piguet^1,2^, Muireann Irish^1,2^*

**Author affiliations:**

1 Brain and Mind Centre, The University of Sydney, Sydney, New South Wales, Australia

2 School of Psychology, The University of Sydney, Sydney, New South Wales, Australia

3 Central Clinical School, The University of Sydney, Sydney, New South Wales, Australia

4 Neuropsychology Unit, Royal Prince Alfred Hospital, Sydney, New South Wales, Australia

5 Macquarie University Health, Macquarie University, Sydney, New South Wales, Australia

6 Department of Neurology, Gosford Hospital, Gosford, New South Wales, Australia

7 School of Medicine and Public Health, Central Coast Clinical School, University of Newcastle, Newcastle, New South Wales, Australia

8. Department of Clinical Neurosciences, Memory and Cognition Clinic, Royal Prince Alfred Hospital, Sydney, New South Wales, Australia

***Correspondence**

Professor Muireann Irish, Brain and Mind Centre, The University of Sydney, Sydney, New South Wales 2050, Australia. E-mail: [muireann.irish@sydney.edu.au](mailto:muireann.irish@sydney.edu.au)

**Files included:**

**Supplementary Fig. 1** Missingness map of demographic, clinical, and cognitive variables among patients with complete ZBI data (n = 358) prior to multiple imputation.

**Supplementary Fig. 2** Associations between (A) behavioural rigidity and (B) apathy and carer burden in the primary analytic cohort (N = 358).

**Supplementary Fig. 3** Association between behavioural rigidity and carer burden in the clinically probable FTLD group (N = 230).

**Supplementary Fig. 4** Associations between (A) abnormal behaviour and (B) apathy and carer burden in the clinically probable AD group (N = 128).

**Supplementary Fig. 5** Association between longitudinal changes in mood symptoms (e.g., irritability and agitation) and longitudinal changes in carer burden in the primary longitudinal analytic cohort (N = 172).

**Supplementary Fig. 6** Associations between behavioural and psychological symptoms (assessed by CBI-R), global cognition (assessed by ACE-III), and carer burden (assessed by ZBI) using imputed data (ZBI, N = 432).

**Supplementary Fig. 7** Associations between longitudinal changes (i.e., T2−T1) in BPSD (assessed by CBI-R) and longitudinal changes (i.e., T2−T1) in carer burden (assessed by ZBI) using imputed data (ZBI, N = 175).

**Supplementary Fig. 8** Associations between behavioural and psychological symptoms (assessed by CBI-R), global cognition (assessed by ACE-III), and carer burden (assessed by ZBI) in the FTLD group with complete ZBI data.

**Supplementary Table 1** Proportion of missing data across the seven BPSD domains by diagnostic group in the primary analytic cohort (N = 358).

**Supplementary Table 2** Carer burden, global cognition, and behavioural and psychological symptoms in the primary analytic cohort (N = 358).

**Supplementary Table 3** Demographic characteristics of carers in the full dementia cohort.

**Supplementary Fig. 1** Missingness map of demographic, clinical, and cognitive variables among patients with complete ZBI data (n = 358) prior to multiple imputation. A missing CBI-R total score does not necessarily indicate missing BPSD data, as participants may have complete data for the seven BPSD domains of interest but were missing data in the other three CBI-R domains. BPSD, Behavioural and psychological symptoms of dementia; ACE-III, Total score of Addenbrooke’s Cognitive Examination-III; FRS, Logit score of Frontotemporal Dementia Functional Rating Scale; ZBI, Total score of Zarit Burden Interview; CBI-R, Total score of Cambridge Behavioural Inventory-Revised.

**Supplementary Fig. 2** Associations between (A) behavioural rigidity and (B) apathy and carer burden in the primary analytic cohort (N = 358). bvFTD, behavioural variant frontotemporal dementia; SD left, left-sided semantic dementia; SD right, right-sided semantic dementia; PNFA, progressive nonfluent aphasia; LPA, logopenic progressive aphasia; CBS, corticobasal syndrome; PSP, progressive supranuclear palsy; AD, Alzheimer’s disease. ZBI, Zarit Burden Interview.

**Supplementary Fig. 3** Association between behavioural rigidity and carer burden in the clinically probable FTLD group (N = 230). FTLD, frontotemporal lobar degeneration; bvFTD, behavioural variant frontotemporal dementia; SD left, left-sided semantic dementia; SD right, right-sided semantic dementia; PNFA, progressive nonfluent aphasia; CBS, corticobasal syndrome; PSP, progressive supranuclear palsy. ZBI, Zarit Burden Interview.

**Supplementary Fig. 4** Associations between (A) abnormal behaviour and (B) apathy and carer burden in the clinically probable AD group (N = 128). LPA, logopenic progressive aphasia; AD, Alzheimer’s disease. ZBI, Zarit Burden Interview.

**Supplementary Fig. 5** Association between longitudinal changes in mood symptoms (e.g., irritability and agitation) and longitudinal changes in carer burden in the primary longitudinal analytic cohort (N = 172). bvFTD, behavioural variant frontotemporal dementia; SD left, left-sided semantic dementia; SD right, right-sided semantic dementia; PNFA, progressive nonfluent aphasia; LPA, logopenic progressive aphasia; CBS, corticobasal syndrome; PSP, progressive supranuclear palsy; AD, Alzheimer’s disease.

**Supplementary Fig. 6** Associations between behavioural and psychological symptoms (assessed by CBI-R), global cognition (assessed by ACE-III), and carer burden (assessed by ZBI) using imputed data (ZBI, N = 432). Models were adjusted for patient age, sex, education, illness duration, disease severity, and diagnostic category. Standardised regression coefficients from multivariate linear regression models predicting carer burden are shown for A) full dementia cohort, C) the FTLD group, and E) the AD group. Relative importance values from the same models are shown for B) full dementia cohort, D) the FTLD group, and F) the AD group. Floating lines depict 95% confidence intervals (CIs), and an association is supported when the CI does not include zero. Higher relative importance value represents larger importance in the regression model. Lower ACE-III scores reflect poorer global cognition, while higher CBI-R and ZBI scores indicate greater symptom severity. CBI-R, Cambridge Behavioural Inventory-Revised; ACE-III, Addenbrooke’s Cognitive Examination–Third edition; ZBI, Zarit Burden Interview.

FTLD, clinically probable frontotemporal lobar degeneration; AD, clinically probable Alzheimer’s disease. The FTLD group comprised clinical syndromes typically associated with FTLD pathology, including behavioural variant frontotemporal dementia (bvFTD), left-sided semantic dementia (SD-left), right-sided semantic dementia (SD-right), progressive nonfluent aphasia (PNFA), corticobasal syndrome (CBS), and progressive supranuclear palsy (PSP). The AD group comprised clinical syndromes typically associated with AD pathology, including typical AD and logopenic progressive aphasia (LPA).

**Supplementary Fig. 7** Associations between longitudinal changes (i.e., T2−T1) in BPSD (assessed by CBI-R) and longitudinal changes (i.e., T2−T1) in carer burden (assessed by ZBI) using imputed data (ZBI, N = 175). Models were adjusted for baseline patient age, sex, education, illness duration, disease severity, and diagnostic category. Standardised regression coefficients from multivariate linear regression models predicting longitudinal change in carer burden are shown for A) the full dementia cohort and C) the FTLD group. Relative importance values from the same models are shown for B) the full dementia cohort and D) the FTLD group. Floating lines depict 95% confidence intervals (CIs), and an association is supported when the CI does not include zero. Higher relative importance values represent stronger contributions to the regression model. CBI-R, Cambridge Behavioural Inventory–Revised; ZBI, Zarit Caregiver Burden Interview. FTLD, clinically probable frontotemporal lobar degeneration. The FTLD group comprised clinical syndromes typically associated with FTLD pathology, including behavioural variant frontotemporal dementia (bvFTD), left-sided semantic dementia (SD-left), right-sided semantic dementia (SD-right), progressive nonfluent aphasia (PNFA), corticobasal syndrome (CBS), and progressive supranuclear palsy (PSP).

**Supplementary Fig. 8** Associations between behavioural and psychological symptoms (assessed by CBI-R), global cognition (assessed by ACE-III), and carer burden (assessed by ZBI) in the FTLD group with complete ZBI data. Models were adjusted for patient age, sex, education, illness duration, disease severity, and diagnostic category. Standardised regression coefficients from multivariate linear regression models predicting carer burden in the FTLD group are shown after (A) excluding bvFTD and (C) excluding left- and right-sided SD. Relative importance values from the same models are shown after (B) excluding bvFTD and (D) excluding left- and right-sided SD. Floating lines depict 95% confidence intervals (CIs), and an association is supported when the CI does not include zero. Higher relative importance value represents larger importance in the regression model. Lower ACE-III scores reflect poorer global cognition, while higher CBI-R and ZBI scores indicate greater symptom severity. CBI-R, Cambridge Behavioural Inventory-Revised; ACE-III, the Addenbrooke’s Cognitive Examination–Third edition; ZBI, Zarit Burden Interview. FTLD, clinically probable frontotemporal lobar degeneration. The FTLD group comprised clinical syndromes typically associated with FTLD pathology, including behavioural variant frontotemporal dementia (bvFTD), left-sided semantic dementia (SD-left), right-sided semantic dementia (SD-right), progressive nonfluent aphasia (PNFA), corticobasal syndrome (CBS), and progressive supranuclear palsy (PSP).

**Supplementary Table 1.** Proportion of missing data across the seven BPSD domains by dementia subtype in the primary analytic cohort (N = 358).

|  | bvFTD  (n = 85) | SD left (n = 41) | SD right  (n = 18) | CBS  (n = 28) | PNFA  (n = 36) | PSP  (n = 22) | AD  (n = 90) | LPA  (n = 38) |
| --- | --- | --- | --- | --- | --- | --- | --- | --- |
| Abnormal behaviour | 6 (7.1%) | 2 (4.9%) | 0 (0%) | 1 (3.6%) | 0 (0%) | 1 (4.5%) | 2 (2.2%) | 0 (0%) |
| Mood disturbance | 4 (4.7%) | 1 (2.4%) | 0 (0%) | 0 (0%) | 1 (2.8%) | 0 (0%) | 0 (0%) | 0 (0%) |
| Abnormal belief | 1 (1.2%) | 0 (0%) | 0 (0%) | 0 (0%) | 0 (0%) | 0 (0%) | 1 (1.1%) | 0 (0%) |
| Eating habits | 2 (2.4%) | 1 (2.4%) | 0 (0%) | 0 (0%) | 0 (0%) | 0 (0%) | 2 (2.2%) | 2 (5.3%) |
| Sleep disturbance | 6 (7.1%) | 1 (2.4%) | 0 (0%) | 1 (3.6%) | 1 (2.8%) | 0 (0%) | 1 (1.1%) | 1 (2.6%) |
| Behavioural rigidity | 3 (3.5%) | 1 (2.4%) | 0 (0%) | 0 (0%) | 3 (8.3%) | 1 (4.5%) | 2 (2.2%) | 1 (2.6%) |
| Apathy | 1 (1.2%) | 2 (4.9%) | 0 (0%) | 1 (3.6%) | 2 (5.6%) | 0 (0%) | 2 (2.2%) | 0 (0%) |

Note: Data are presented as number (%) of participants with missing data for each BPSD domain. BPSD, behavioural and psychological

symptoms of dementia; bvFTD, behavioural variant frontotemporal dementia; SD left, left-sided semantic dementia; SD right, right-sided

semantic dementia; PNFA, progressive nonfluent aphasia; LPA, logopenic progressive aphasia; CBS, corticobasal syndrome; PSP, progressive

supranuclear palsy; AD, Alzheimer’s disease

**Supplementary Table 2** Carer burden, global cognition, and behavioural and psychological symptoms by dementia subtype in the primary analytic cohort (N = 358).

|  | **bvFTD** | | **SD left** | **SD right** | **PNFA** | **LPA** | **CBS** | **PSP** | **AD** |
| --- | --- | --- | --- | --- | --- | --- | --- | --- | --- |
| Carer burden | | 22.88 (9.99) | 13.83 (10.32) | 20.17 (8.87) | 8.64 (6.49) | 11.79 (8.56) | 10.82 (7.77) | 15.82 (12.07) | 16.17 (9.56) |
| Global cognition | | 77.07 (12.21) | 61.88 (13.85) | 62.61 (14.63) | 76.72 (12.66) | 65.73 (12.67) | 74.18 (12.20) | 77.96 (9.65) | 68.46 (13.21) |
| Behavioural rigidity | | 7.67 (4.39) | 4.88 (4.70) | 8.50 (4.73) | 1.82 (2.42) | 2.11 (2.50) | 2.36 (2.98) | 4.92 (4.55) | 3.82 (3.66) |
| Apathy | | 11.57 (6.13) | 5.41 (5.23) | 10.06 (5.70) | 3.77 (4.19) | 4.37 (4.67) | 5.95 (5.78) | 8.23 (6.23) | 6.08 (4.91) |
| Abnormal behaviour | | 9.03 (5.90) | 4.08 (4.94) | 6.94 (4.21) | 1.39 (1.96) | 2.47 (3.48) | 2.47 (3.60) | 5.35 (6.05) | 3.45 (3.60) |
| Mood disturbance | | 4.44 (2.93) | 3.03 (2.64) | 4.89 (2.49) | 2.19 (2.33) | 2.18 (2.29) | 3.82 (3.15) | 4.05 (3.20) | 3.64 (2.89) |
| Abnormal beliefs | | 0.97 (1.68) | 0.46 (1.40) | 1.22 (1.56) | 0.08 (0.28) | 0.08 (0.36) | 0.21 (0.96) | 0.23 (0.53) | 0.63 (1.46) |
| Eating habits | | 7.04 (4.70) | 2.08 (3.14) | 5.00 (3.63) | 1.00 (1.80) | 1.41 (2.21) | 2.50 (3.49) | 3.55 (3.81) | 2.35 (2.82) |
| Sleep disturbance | | 3.36 (2.42) | 1.56 (1.52) | 3.11 (2.22) | 1.96 (2.17) | 1.81 (1.83) | 2.99 (2.60) | 3.00 (2.14) | 2.61 (2.17) |

Note: bvFTD, behavioural variant frontotemporal dementia; SD left, left-sided semantic dementia; SD right, right-sided semantic dementia; PNFA, progressive nonfluent aphasia; LPA, logopenic progressive aphasia; CBS, corticobasal syndrome; PSP, progressive supranuclear palsy; AD, Alzheimer’s disease. Values are based on imputed data, except for carer burden and global cognition, for which complete observed data were used. Global cognition indexed via the total score on the ACE-III screening tool. BPSD symptoms indexed using the Cambridge Behavioural Inventory-Revised.

**Supplementary Table 3.** Demographic characteristics of carers in the full cohort.

|  | **Full dementia cohort**  **(n = 432)** | **FTLD group^a^**  **(n = 287)** | **AD group^b^**  **(n = 155)** |
| --- | --- | --- | --- |
| Age, years | 60.16 (12.78) | 59.98 (12.48) | 60.49 (13.37) |
| Education, years | 12.20 (4.12) | 12.01 (4.19) | 12.58 (3.97) |
| Sex, male: female | 129:251 | 141:108 | 73:64 |
| Relationship | Total 386 | Total 249 | Total 137 |
| Spouse/partner | 316 | 201 | 115 |
| Child | 46 | 33 | 11 |
| Siblings | 13 | 10 | 3 |
| Friend | 10 | 4 | 6 |
| Other (Mother) | 1 | 1 | 0 |
| Unknown | 46 | 38 | 18 |

Note: Means with standard deviations in parentheses. Missing data were present for some variables and occurred across different individuals. FTLD, clinically probable frontotemporal lobar degeneration; AD, clinically probable Alzheimer’s disease. ^a^FTLD group comprises behavioural variant frontotemporal dementia (bvFTD), left-sided semantic dementia (SD-left), right-sided semantic dementia (SD-right), progressive nonfluent aphasia (PNFA), corticobasal syndrome (CBS), and progressive supranuclear palsy (PSP). ^b^AD group comprises clinically probable AD and logopenic progressive aphasia (LPA).
